# A Review of Advances in Molecular Imaging of Rheumatoid Arthritis: From In Vitro to Clinic Applications Using Radiolabeled Targeting Vectors with Technetium-99m

**DOI:** 10.3390/life14060751

**Published:** 2024-06-12

**Authors:** Muhammad Ali, Viviana Benfante, Domenico Di Raimondo, Riccardo Laudicella, Antonino Tuttolomondo, Albert Comelli

**Affiliations:** 1 Ri.MED Foundation, Via Bandiera 11, 90133 Palermo, Italy; amuhammad@fondazionerimed.com (M.A.); acomelli@fondazionerimed.com (A.C.); 2 Department of Health Promotion, Mother and Child Care, Internal Medicine and Medical Specialties, Molecular and Clinical Medicine, University of Palermo, 90127 Palermo, Italy; domenico.diraimondo@unipa.it (D.D.R.); bruno.tuttolomondo@unipa.it (A.T.); 3 Nuclear Medicine Unit, Department of Biomedical and Dental Sciences and Morpho-Functional Imaging, Messina University, 98124 Messina, Italy; rlaudicella@unime.it; 4 NBFC—National Biodiversity Future Center, 90133 Palermo, Italy

**Keywords:** ^99m^Tc, autoimmune disorder, computed tomography, nuclear medicine, RA, radiopharmaceuticals, scintigraphy, single-photon emission computed tomography, SPECT/CT, synovitis

## Abstract

Rheumatoid arthritis (RA) is a systemic autoimmune disorder caused by inflammation of cartilaginous diarthrodial joints that destroys joints and cartilage, resulting in synovitis and pannus formation. Timely detection and effective management of RA are pivotal for mitigating inflammatory arthritis consequences, potentially influencing disease progression. Nuclear medicine using radiolabeled targeted vectors presents a promising avenue for RA diagnosis and response to treatment assessment. Radiopharmaceutical such as technetium-99m (^99m^Tc), combined with single photon emission computed tomography (SPECT) combined with CT (SPECT/CT), introduces a more refined diagnostic approach, enhancing accuracy through precise anatomical localization, representing a notable advancement in hybrid molecular imaging for RA evaluation. This comprehensive review discusses existing research, encompassing in vitro, in vivo, and clinical studies to explore the application of ^99m^Tc radiolabeled targeting vectors with SPECT imaging for RA diagnosis. The purpose of this review is to highlight the potential of this strategy to enhance patient outcomes by improving the early detection and management of RA.

## 1. Introduction

Rheumatoid arthritis (RA) is a persistent, systemic, inflammatory autoimmune disease that primarily affects the synovial joints, causing damage to cartilage and bone and extra-articular involvement such as the skin, eyes, lungs, digestive system, nervous system, heart, and kidney [1,2]. Common symptoms of RA include morning stiffness lasting for more than 30 min in the affected joint accompanied by swelling, appetite loss and a limited range of motion [3]. Approximately 1% of the global population suffers from RA [4], and women have a threefold higher likelihood of developing RA than men [5,6,7]. The annual expenses of RA are estimated to be $48 billion (2016 US dollars) in United states. These costs include direct healthcare costs as well as indirect costs such as lost productivity and intangible costs to the employer and family [8,9]. According to a recent modeling analysis, the yearly economic burden of RA in Italy was projected to be approximately EUR 2.0 billion in 2015. Direct medical expenses accounted for 45% of the expenditure. Indirect costs accounted for nearly half of the expenditure, around 45%, while direct non-medical expenditures determined the remaining 10% [10].

**Figure 1:**
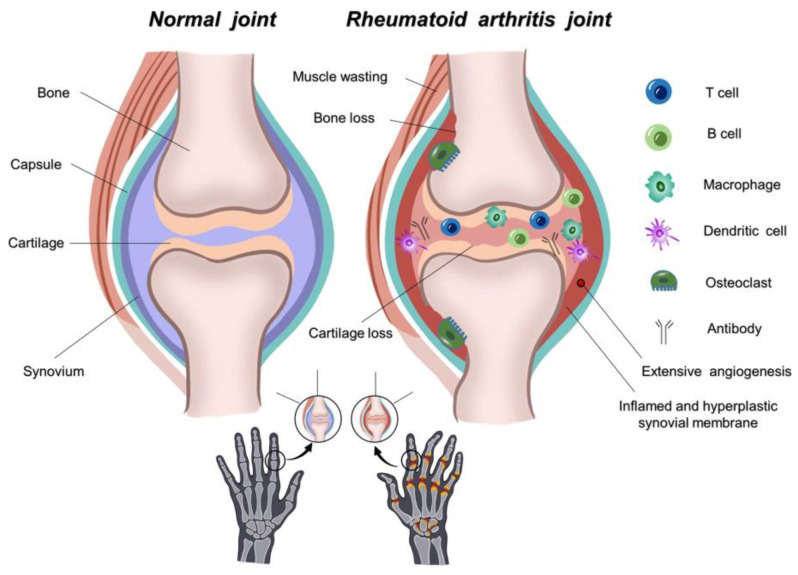
Normal and rheumatoid arthritis joints. Reprinted from open access source (CC BY) [13].

In RA, inflammation of the synovium, called synovitis, leads to hyperplasia of the synovial lining and destruction of the cartilage and bones in the joints [11,12,13]. A healthy synovium consists of 1–2 linings of synoviocytes [14,15,16]. During inflammation, various mononuclear cells, such as T cells, B cells, dendritic cells, plasma cells, mast cells, and macrophages, infiltrate the site of inflammation (Figure 1) [17,18,19,20]; the synovial lining becomes hyperplastic, resulting in expansion of the synovial membrane and the formation of villi [21,22,23]. Macrophage-like synoviocytes produce various proinflammatory cytokines, such as Interleukin (IL)-1, IL-6, and tumor necrosis factor (TNF). Fibroblast-like synoviocytes (FLSs) produce IL-6, leukotrienes and prostaglandins and migrate from joint to joint for disease propagation [12,14,24,25]. Synovial cell proliferation reduces capillary flow, increasing fluid volume in the synovium and resulting in hypoxia at the inflamed site [12,26,27,28,29,30,31]. Due to the increased volume in the synovium, angiogenesis occurs, induced by various factors, such as vascular endothelial growth factor (VEGF) [32,33,34,35].

**Figure 2:**
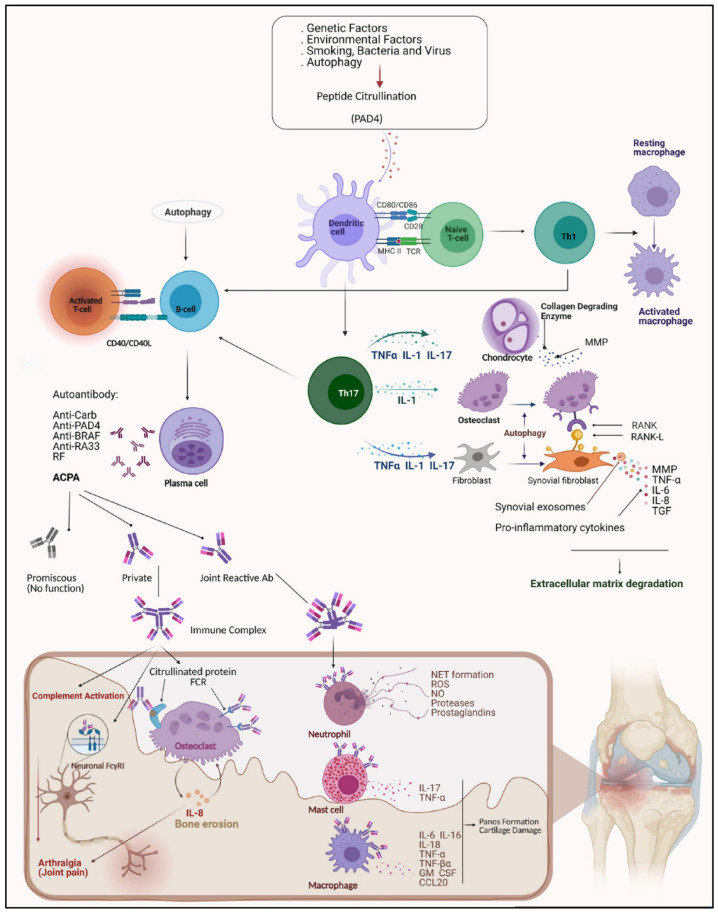
Rheumatoid arthritis etiology. Reprinted from open access source (CC BY) [52].

Bone erosion is attributed to osteoclasts maturation and activation through receptor activation of the nuclear factor-kappa B (RANK) ligand, which is produced by T cells, together with IL-1, IL-6, and TNF produced by FLSs and macrophages in the synovial lining [36,37,38,39,40,41,42]. Osteoclasts release proteases such as cathepsin K, which degrade cartilage and bones. It has also been suggested that anti-citrullinated protein antibodies (ACPAs) interact with citrullinated peptides on osteoclasts, leading to their maturation and activation (Figure 2) [43,44,45,46,47,48,49,50,51].

### 1.1. Molecular Imaging

In molecular imaging, biological processes in living systems can be observed, analyzed, and measured at the molecular by imaging modalities such as ultrasound, magnetic resonance imaging (MRI), positron emission tomography (PET), and single photon emission computed tomography (SPECT) [53,54,55,56,57]. PET and SPECT are sophisticated imaging modalities utilized to visualize biological processes at the molecular level [58,59,60,61]. PET is more sensitive while SPECT is more widely available, less expensive, and has better imaging properties than PET [62]. The estimated cost of SPECT/CT imaging in a clinical setting is around $622 per patient per year [63].

In SPECT imaging, gamma rays emitted by radionuclides are detected using a gamma camera, which is linked with a computer algorithm to transform radioactivity emitted from radionuclides (possessing diagnostic information) into a precise picture of the distribution of radionuclides in the body [58,59,60,64,65]. Most radiopharmaceuticals used in SPECT are single molecules radiolabeled with a gamma-emitting isotope, such as iodine-123, gallium-67, indium-111 or technetium-99m. SPECT provides 3D images that are more sensitive than structural images [66,67]. The radiopharmaceutical agent is usually made of two elements, a carrier or ligand and a radioactive atom, which determines the diagnostic character of the radioactive compound through its nuclear properties. The carrier is crucial for precisely delivering the radionuclide to a biological target [68,69]. Chelators and linkers are used to link ligands and radionuclides; indeed, radionuclides such as ^99m^Tc and ^66/68^Ga require chelators for stability, while linkers are used optionally to increase radionuclides pharmacokinetics and binding affinity [70,71].

Clinical research radiotracers should meet the following criteria: short half-life, 100–600 keV monochromatic gamma ray emission for diagnostic applications; low toxicity, high stability, and high specificity; low plasma protein binding affinity; and rapid elimination [62,72,73].

### 1.2. Technetium-99m Overview

The radionuclide isotope technetium-99m (^99m^Tc) has been widely utilized in imaging and diagnostic procedures [74,75]. ^99m^Tc is the FDA- and EMA-approved and most common radioactive isotope tracer used in SPECT for imaging of the kidneys, brain, thyroid, heart, liver, gallbladder, spleen, salivary and lachrymal glands, sentinel nodes, and bone marrow [76]. The short half-life of 99mTc makes it an ideal radionuclide for radiation exposure [74]. The radioisotope technetium-99m emits gamma radiation (140.5 keV) and can be detected noninvasively on the outside of the body using imaging SPECT systems [77].

Technetium-99m can be introduced into bioactive molecules using inorganic technetium functional groups, which are synthesized in a physiological solution with a decreased oxidation state of technetium and labile coordination sites that are easily exploited to incorporate the desired bioactive component [65].

**Figure 3:**
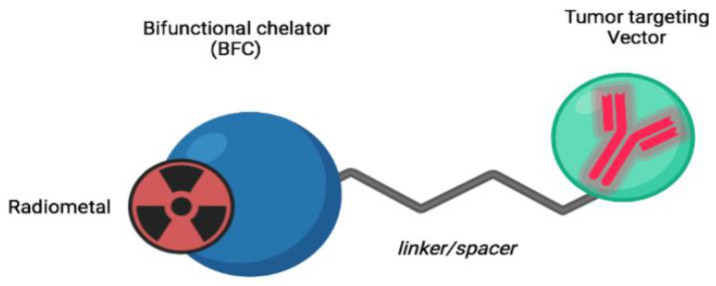
Metal fragment strategy for bioactive molecule labeling. Reprinted from open access source (CC BY-NC-ND 4.0) [79].

When combined with a suitable set of coordinating atoms, the metal fragments shown in Figure 3 offer a valuable method for attaching a physiological moiety to a technetium-99m [65]. The radioactive metal fragment and a chelating group are attached to the bioactive molecule via a spacer group. Due to the strong affinity of the precursor metal fragment for particular binding sites on the ligand, two properly chosen molecular building blocks can form a conjugate complex [78].

## 2. Potential Targets for the Molecular Imaging of Rheumatoid Arthritis

**Figure 4:**
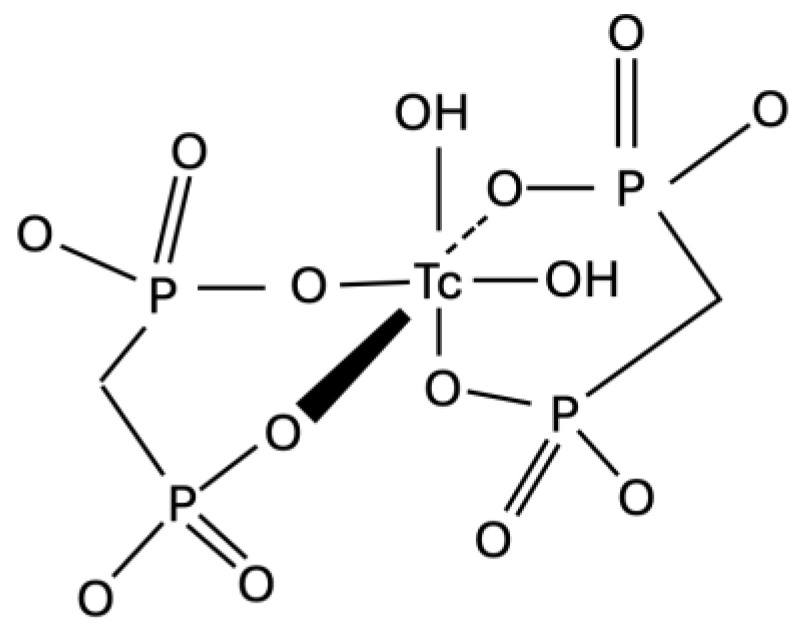
Structure image of ^99m^Tc-MDP.

99mTc-labeled diphophonates can be used to visualize active synovitis in RA joints and detect areas of increased bone activity potentially associated with articular bone reaction/destruction (Figure 4) [80].

However, a three-phase bone scan with ^99m^Tc-diphosphonates is suboptimal for predicting active inflammation in RA [81,82]. ^99m^Tc-diphosphonates can be used to observe postsurgical complications, fractures, or orthopedic hardware complications [83,84].

To assess active inflammation in RA, ^99m^Tc can be also labeled with ligands or receptors overexpressed in RA such as integrin αvβ3, antibodies, cytokines, folate receptor, translocator protein (TSPO), or the macrophage mannose receptor (MMR) [77,85,86,87,88,89,90,91,92]. This targeted approach allows for a more accurate assessment of active inflammation in RA.

### 2.1. Monoclonal Antibodies

**Figure 5:**
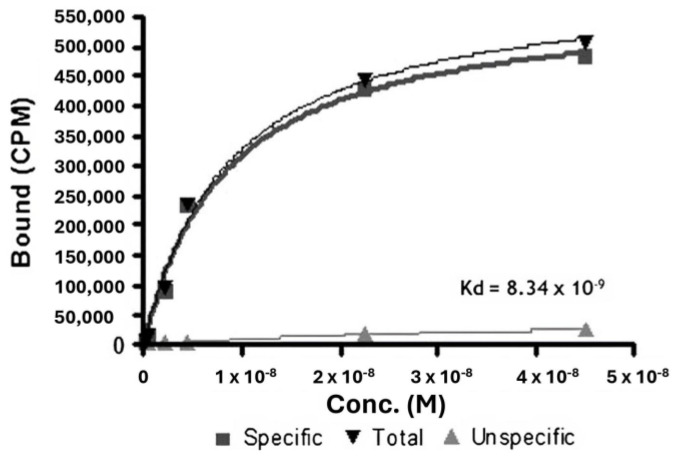
^99m^Tc-labeled rituximab binding curve in RAJI cells. Reprinted from open-access source (CC BY) [103].

In 2006, rituximab was approved for the treatment of RA [93,94]. Rituximab is a genetically engineered monoclonal antibody to CD20 antigen [95]. CD20 antigen is expressed on B lymphocytes [96]. B lymphocytes play an important role in RA pathogenesis. B lymphocytes release proinflammatory cytokines, ACPAs and rheumatoid factors (RFs) [97]. B lymphocytes also express costimulatory molecules which facilitate T-cell activation [98], which involves stimulating and activating fibroblasts and macrophages [99,100,101,102]. Rituximab binds to the CD antigen, disrupts signaling pathways, and triggers apoptosis via various mechanisms, such as Antibody-dependent cellular cytotoxicity (ADCC) and Complement-dependent cytotoxicity (CDC). In one study, 20 patients with various chronic inflammatory autoimmune disorders received rituximab, labeled with ^99m^Tc. At 6 and 20 h post-injury, whole-body scintigraphic images were acquired. Rapid absorption by the spleen and renal excretion of radioactivity was observed by [^99m^Tc]Tc-rituximab scintigraphy. Patients with rheumatoid arthritis who had inflamed joints at 6 h post-infection displayed varying degrees of uptake. These findings demonstrated that in individuals with autoimmune illnesses, this method can be used to evaluate B lymphocyte infiltration into impacted organs. The RAJI cell line was used to determine the binding affinity of [99mTc]Tc-rituximab to CD20 receptors. The binding affinity was greater than that of native rituximab, which has a Kd of 5.2 nM. This finding implies that rituximab maintained the ability to selectively bind to CD20 receptors even after technetium-99m radiolabeling. (Figure 5) [103].

In another study, T-cell traffic and lymphocytic infiltration of organs affected by autoimmune illness were imaged using a radiolabeled anti-CD3 antibody. Visilizumab, [^99m^Tc]Tc-succinimidyl-6-hydrazinonicotinate hydrochloride (SHNH), was evaluated against HuT78 cells and BALB/c xenografted mice. The findings showed that HYNIC maintained its biochemical integrity and in vitro binding activity to CD3-positive cells while exhibiting high specific activity (SA; 10,360–11,100 MBq/mg) and labeling efficiency (>90%). The in vivo targeting experiment revealed that visilizumab labeled with 99mTc can be used for T lymphocyte trafficking and lymphocytic infiltration of tissues and organs [104].

### 2.2. Macrophages

Macrophages play a crucial role in RA pathogenesis by actively producing various substances, e.g., tumor necrosis factor (TNF), proinflammatory or regulatory cytokines, growth factors, chemokines, metalloproteinases, and neopterin [105,106,107]. During inflammation, macrophages concentrate at the junction of the synovial cartilage pannus. Macrophages are classified into two types: activated macrophages (M1) and alternatively stimulated macrophages (M2). M1 macrophages are proinflammatory, while M2 macrophages are anti-inflammatory. [108,109,110]. Furthermore, M1 macrophages also express major histocompatibility complex class II (MHC II), CD68, CD80, and CD86 [48,111]. M2 macrophages express various specific receptors and chemokines, such as CD206, CD163, CD209, FIZZ1, and Ym1/2, which are essential for the phagocytosis and scavenging of mannose and galactose [112,113].

In one study, it was determined whether administering tilmanocept subcutaneously (SC) with a ^99m^Tc label could identify macrophage-mediated inflammation in RA patients but not in healthy control (HC) subjects. [^99m^Tc]Tc-tilmanocept was injected into 18 RA patients. Patients were imaged via a whole-body planar scan at 2–3 h and 4–6 h post-injection. After the whole-body scan, 5 min planar images of both hands were obtained. The results revealed that [^99m^Tc]Tc-tilmanocept was significantly taken up by the affected joint [114].

Kardan et al. highlighted the potential use of intravenous administration of [^99m^Tc]Tc-tilmanocept in monitoring RA progression in the first-in-human phase I/phase II clinical study. The study involved 33 patients with active RA and six healthy volunteers, with various dose combinations of ^99m^Tc administered intravenously. The imaging results revealed that [^99m^Tc]Tc-tilmanocept can specifically localize inflamed joints, offering a foundation for a noninvasive method to monitor disease activity in macrophage-driven inflamed joints with RA [115].

**Figure 6:**
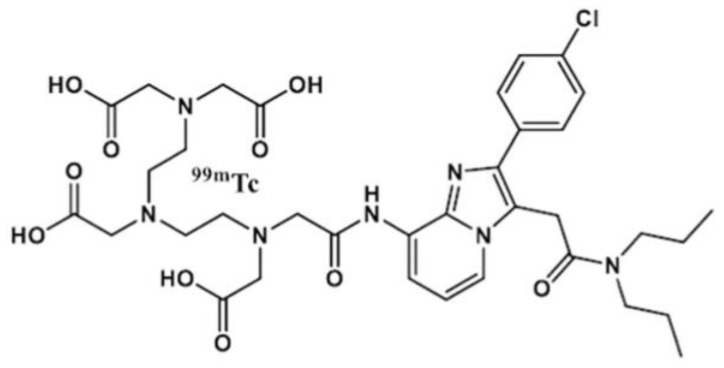
Chemical structure of [^99m^Tc]Tc-DTPA-CB86. Reprinted from open access source (CC BY). [116].

**Figure 7:**
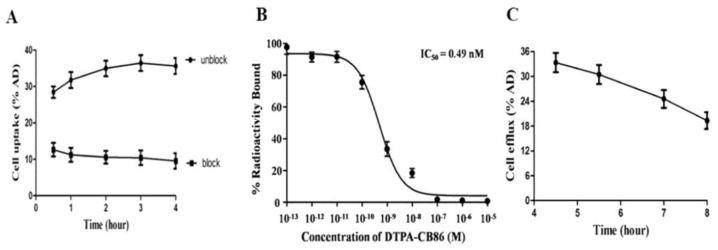
Binding assays of [^99m^Tc]Tc-DTPA-CB86 in RAW264.7 cells. (**A**) Cellular uptake assay. (**B**) Receptor saturation assay. (**C**) Cellular efflux assay. Reprinted from open-access source (CC BY-NC-ND 4.0) [116].

**Figure 8:**
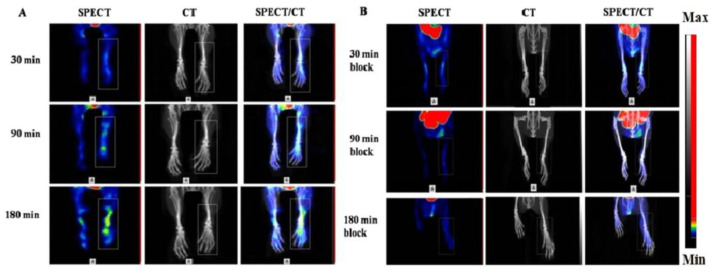
SPECT/CT imaging of [^99m^Tc]Tc-DTPA-CB86 in RA model rats coinjected with 0 μg (unblock, (**A**)) or 300 μg (block, (**B**)) of DTPA-CB86 at various intervals after injection. Reprinted from open source (CC BY) [116].

The mitochondrial membrane translocator protein (TSPO) present on the macrophage surface can detect and quantify RA. In one study, ^99m^Tc-labeled DTPA-CB86 (Figure 6) was used to analyze TSPO binding affinity in RAW264.7 cells. Figure 7A shows the [^99m^Tc]Tc-DTPA-CB86 cell uptake ratio. In RAW264.7 cells, the peak accumulation of [^99m^Tc]Tc-DTPA-CB86 occurred 180 min later, reaching 36.45 ± 2.18% of the applied activity. A receptor saturation test confirmed the binding of [^99m^Tc]Tc-DTPA-CB86 to TSPO, revealing an IC50 value of 0.49 nM (Figure 7B). Moreover, cell efflux tests demonstrated that [^99m^Tc]Tc-DTPA-CB86 displayed robust cell retention in the cell line, with approximately 13.99% efflux observed after 4.5 to 8 h of incubation (Figure 7C). These findings suggest that the labeling procedure did no affect the ability of CB86 to bind selectively to TSPO. [^99m^Tc]Tc-DTPA-CB86 rapidly accumulated in the inflamed ankle (Figure 8). After 180 min of administration, [^99m^Tc]Tc-DTPA-CB86 uptake in the inflamed ankle was 2.35 ± 0.10%, which was significantly increased than that in normal tissues using SPECT [116].

In another study, ^99m^Tc-labeled mannosylated dextran or [^99m^Tc]Tc-(CO)3-DCM20 was used to target MMRs for early detection of RA in a mouse model of collagen induced arthritis (CIA). 2-Deoxy-2-18F-fluoro-D-glucose ([^18^F]FDG) was used as a control. The results revealed that [^99m^Tc]Tc-(CO)3-DCM20 accumulated more strongly in the hindpaws, forepaws, and knee joints of CIA mice than in control mice. The radioactivity level of [^99m^Tc]Tc-(CO)3-DCM20 was significantly correlated with the paw clinical score [117].

**Figure 9:**
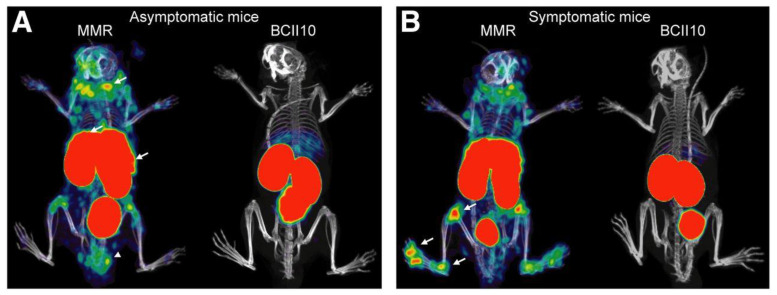
MMR expression in arthritic joints can be detected via in vivo imaging using MMR-specific nanobodies. Micro-CT scans were followed by SPECT imaging. (**A**) Mice that were immunized but did not exhibit clinical arthritis symptoms were injected with labeled MMR nanobodies or BCII10 control nanobodies. (**B**) Mice injected with labeled MMR nanobodies or BCII10 control nanobodies were symptomatic of arthritis in both hind limbs. Reprinted from open access source (CC BY 4.0) [53].

Put et al. used the radiolabeled MMR-targeting nanobodies to detect CD11b+F4/80+ macrophages in the inflamed joints of mice (Figure 9). This observation provided a means of objectively measuring inflammation and further understanding arthritis pathophysiology [53].

### 2.3. Vascular Endothelial Growth Factor

Vascular endothelial growth factor (VEGF) is a member of the mammalian peptide family [118]. These glycoproteins can generate dimeric forms by creating disulfide bridges between two monomers using a particular sequence of cysteines [119]. Each VEGF family member is a glycosylated peptide monomer that must undergo homodimerization or heterodimerization to activate its biological activity [120]. The best-studied member of the VEGF family, VEGF-A (also known as VEGF), is found in several isoforms (e.g., VEGF-A121, VEGF-A145, VEGF-A165, VEGF-A183, VEGF-A189, and VEGF-A206) as a result of alternative splicing of mRNAs produced during the transcription of the human gene VEGFA [121,122,123].

Activated platelets, fibroblasts, lymphocytes, and macrophages also produce VEGF-A glycoproteins [124,125]. Hypoxia is the primary factor that triggers the transcription of the mRNA that encodes VEGF-A [68,126,127,128]. In contrast to hypoxia, normal conditions rigorously control the concentration of HIF-1 in cells. Several hormones, particular growth factors, and cytokines (including interleukin 1b, IL-1b, and tumor necrosis factor alpha [TNF]), among others, are also crucial cellular stimulators of VEGF-A [129].

**Figure 10:**
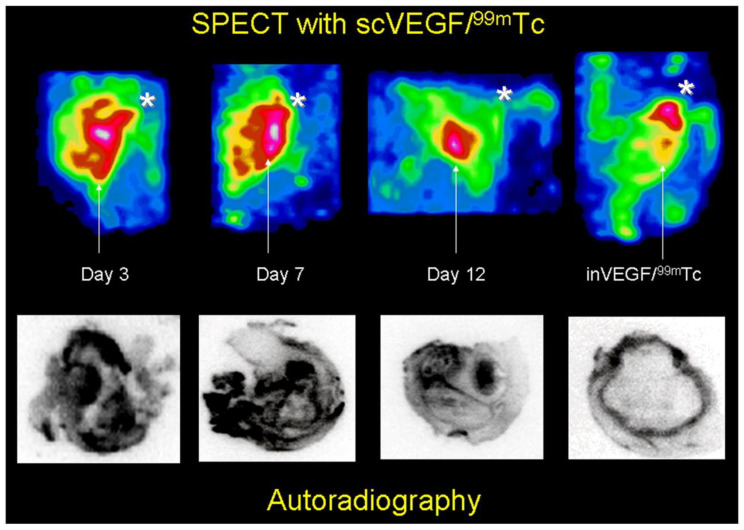
[^99m^Tc]Tc scVEGF SPECT of sterile right thigh abscess. Reprinted from open access source (CC BY 4.0) [130].

Levashova et al. tested whether the labeled form of VEGF could be used to image VEGF receptors in a mouse model [130]. Mice were administered turpentine to induce thigh inflammation. After a few days, the mice were treated with Tc-hydroxynonicotinic acid-single-chain Cys-tagged vascular endothelial growth factor ([^99m^Tc]Tc-inVEGF) and examined via SPECT imaging (Figure 10). SPECT imaging revealed high [^99m^Tc]Tc-inVEGF uptake in the thigh region. It was concluded that the high uptake of scVEGF is linked to the increased expression of VEGFR-2, which is involved in angiogenesis [130].

Galli et al. used a human VEGF165 analog radiolabeled with ^99m^Tc to assess VEGFR expression in HUVECs and in a mouse model (ARO, HT29, and K1). In vitro, [^99m^Tc]Tc-VEGF was shown to bind to HUVECs, and in vivo, [^99m^Tc]Tc-VEGF was shown to bind to xenograft tumors in mice (ARO, K1, and HT29). Comparison of in vivo data with immunohistochemical analysis of excised tumors revealed an inverse correlation between [^99m^Tc]Tc-VEGF165 uptake and histologically detected VEGF [131].

### 2.4. Integrins

**Figure 11:**
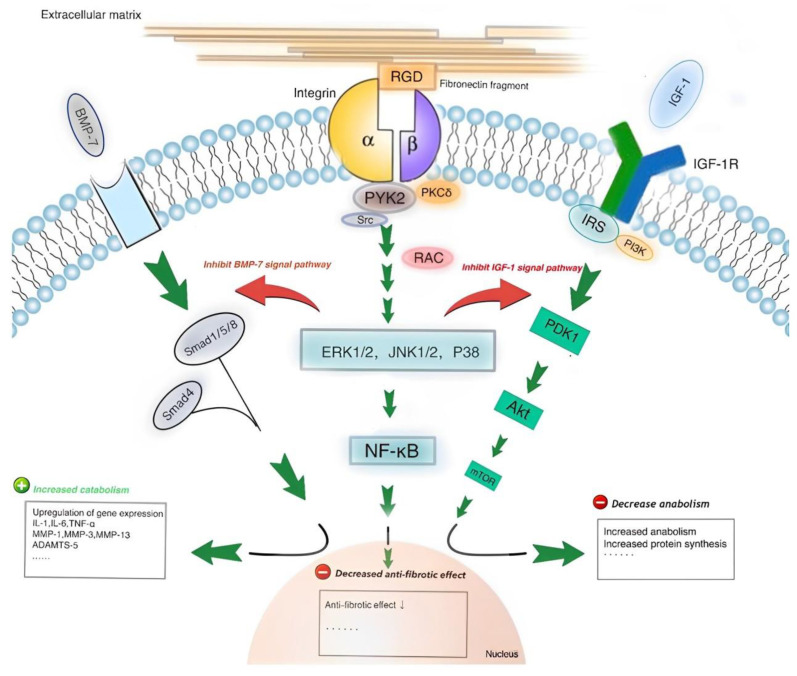
The pathway by which RGD-containing fibronectin fragments induce cartilage damage and proteoglycan loss. Reprinted from open-access source (CC BY 4.0) [142].

Integrins are heterodimers, glycoprotein transmembrane receptors consisting of the α and β subunits with binding sites for the Extracellular matrix (ECM) [132,133]. Upon binding to the ECM, integrins remodel the ECM by upregulating the extrusion of various proteases [134]. Integrins regulate various cellular functions, such as motility, survival, invasion, and inflammation [135,136,137,138,139]. The binding of αvβ3 and α5β1 to Arg-Gly-Asp (RGD) integrins plays an essential role in RA pathogenesis [140,141,142,143]. Their role in angiogenesis is to facilitate EC migration and survival [144]. A cell recognition pattern known as the RGD sequence is present in ECM proteins, such as vitronectin, fibrinogen, and fibronectin [145]. These proteins are required for interactions with integrins. αvβ3 and α5β are known to be as fibronectin receptors. Moreover, αvβ3 binds to osteo-pontin, fibronectin, bone sialoprotein, and vitronectin (Figure 11) [89]. The discovery of the RGD motif has led to the development of numerous peptidic and nonpeptidic integrin ligands with varying degrees of specificity. Integrins exhibit distinct responses when interacting with different RGD-containing ECM proteins, recognizing them individually. Using RGD-based ligands for noninvasive molecular imaging assessment is useful for studying angiogenesis in RA patients [146].

[^99m^Tc]Tc-RGD binding affinity was evaluated in a study using a single HUVEC cell line. αVβ3 integrins were antagonistically treated with radiolabeled RGD peptides, with RGE (Arg-Gly-Glu) serving as the control. Radiolabeled RGD/E binding was assessed at concentrations ranging from picomolar to nanomolar after an hour at 4 °C. ^99m^Tc cell accumulation was up to 16 times greater in the RGD group than in the RGE group. To verify the specificity, 50% of ^99m^Tc-labeled RGD binding to cells was inhibited by 7 nM native cyclized RGD. SE HPLC revealed that the percentage of ^99m^Tc-labeled RGD that bound to the isolated αVβ3 integrin protein increased as the integrin concentration increased [147].

**Figure 12:**
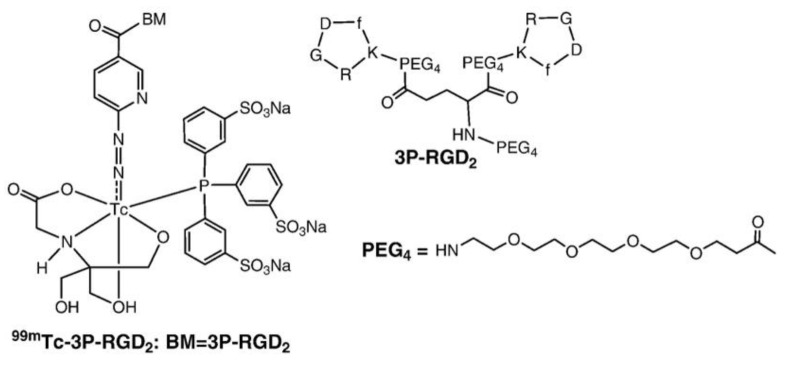
Structural image of [^99m^Tc]Tc-3PRGD2. Reprinted from [149] with permission Copyright 2016. Elseiver.

**Figure 13:**
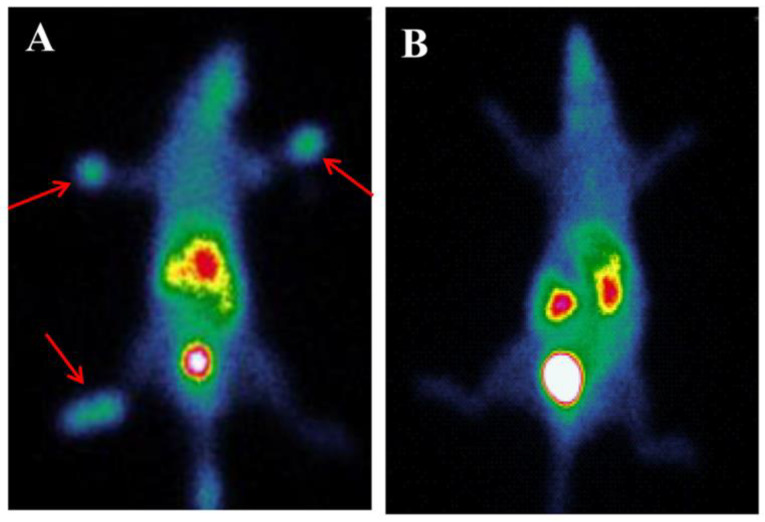
[^99m^Tc]Tc-3PRGD2 imaging of a rat. (**A**) Before bevacizumab treatment. (**B**) After bevacizumab treatment. Reprinted from open access [148].

**Figure 14:**
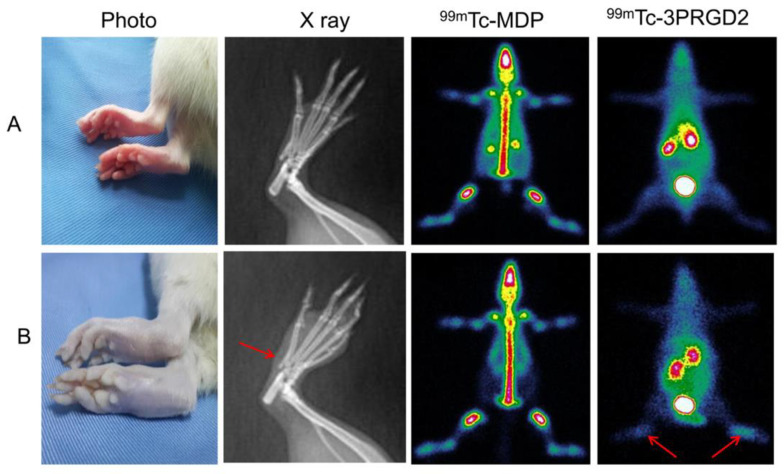
Imaging of [^99m^Tc]Tc-3PRGD2, X-ray and [^99m^Tc]Tc-MDP bone scans. (**A**) Control. (**B**) Rheumatoid arthritis-affected rat. The arrow shows that rheumatoid arthritis is associated with an aberrant increase in [^99m^Tc]Tc-3PRGD2 uptake. Reprinted from open access [148].

Maraciclatide (NC100692 or diamine dioxime-Lys-Cys-Arg-Gly-Asp_Cyc-Phe-Cys-polyethylene glycol) is a cyclic peptide that contains an RGD tripeptide sequence held in a specific conformation by a disulfide moiety and one thioether bridge Cyclic RGD peptides bind to vitronectin receptors. Maraciclatide radiolabelled with technetium-99m was used as in vivo marker for vitronectin integrin receptor expression. In one study, [^99m^Tc]Tc-3PRGD2 (Figure 12) scintigraphy demonstrated increased the uptake of tracers in the joints of arthritic model rats compared to those of untreated controls. Compared with those in the controls, the expression levels of αvβ3 and CD31 were elevated in arthritic joint tissue from the rats. Bevacizumab administration ameliorated arthritis severity and reduced radiotracer uptake in the affected joints (Figure 13). This tracer has been used for identifying early synovial angiogenesis in RA patients. However, [^99m^Tc]Tc-MDP bone scanning could not differentiate between healthy controls and early-stage RA patients (Figure 14) [148].

**Figure 15:**
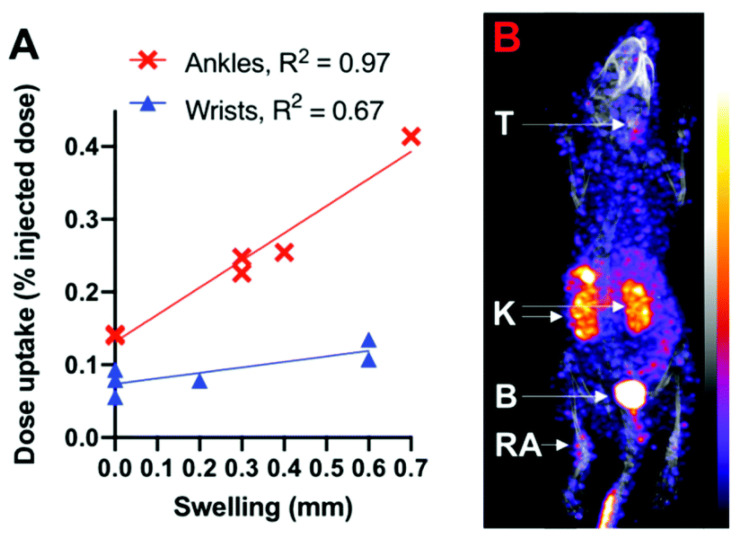
(**A**) Accumulation of radioactivity in the ankles and wrists correlates with joint swelling. (**B**) SPECT/CT image of accumulation of [[^99m^Tc]Tc-O_2_(DP-RGD)2] + in an arthritic ankle (RA). B = bladder, K = kidneys, T = thyroid. Reprinted from open source [150].

In one study, the compound 2,3-bis(diphenylphosphino)maleic anhydride (BMA), also known as diphosphine, was combined with the cyclic peptide Arg-Gly-Asp-Dphe-Lys (RGD), and labeled with ^99m^Tc to specifically target the αvβ3-integrin receptor. This process led to the development of the initial diphosphine–peptide conjugate called DP-RGD. To create the radiotracer [[^99m^Tc]Tc-O_2_(DP-RGD)2]+, DP-RGD was combined with [^99m^Tc]Tc-O_4_. This radiotracer was introduced to mice induced by RA. The RA model demonstrates heterogeneity, displaying varying degrees of arthritis and symptomatic swelling among mice, and even within the joints of the same animal. Analyzing SPECT images obtained one-hour post-injection revealed a correlation between ^99m^Tc radioactivity accumulation and concentration in wrists and ankles (Figure 15) [150].

### 2.5. Somatostatin

**Figure 16:**
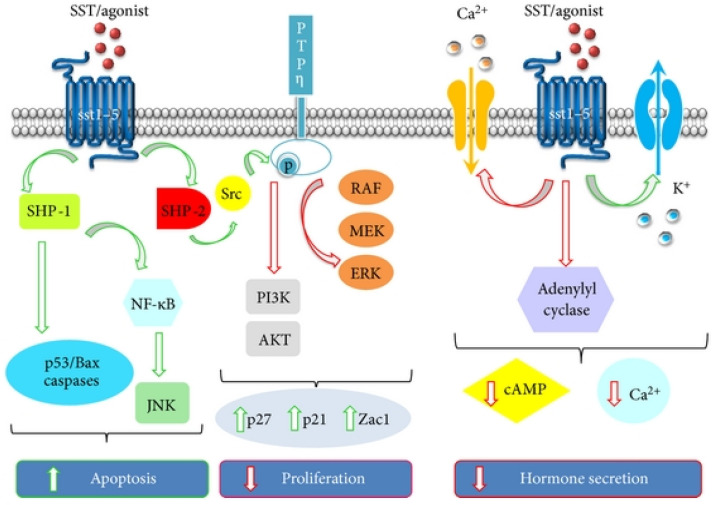
Signaling pathways induced by somatostatin receptor activation. Reprinted from open access source (CC BY) [164].

Somatostatin (SST) is a naturally occurring short cyclic peptide which inhibits cellular proliferation and exocrine secretion, and induces apoptosis [151,152]. Five G protein-linked receptors (SSTR 1, 2, 3, 4, and 5) mediate the actions of somatostatin and its analogs [153,154]. Because of their inhibitory effects, these receptors control various signal transduction pathways (Figure 16). The modulatory effects of somatostatin and its analogs on the immunological response and its antiproliferative, antiangiogenic, and analgesic effects have been studied [155,156,157,158]. In various physiological and pathological circumstances, lymphocytes and vascular endothelium express SSTRs 2, 3, and 5. [152,159,160]. Many studies have suggested that somatostatin (SST) plays a role in angiogenesis. Somatostatin analogs (such as octreotide) have been shown to prevent experimental angiogenesis because they specifically express SST2 in angiogenic sprouts of endothelial cells (HUVECs) in vitro [161,162,163]. It has been demonstrated that somatostatin agonists efficiently reduce VEGF levels and proangiogenic activity in RA patients. Additionally, the angiogenic cascade specifically upregulates SSTR 2. SSTR 2A and 2B are the two receptor isoforms. In mouse arthritis models, the isotype SSTR 2A is highly expressed during the inflammatory response. In synovial biopsies from RA patients, SSTR 2A has been found in the synovial venule endothelium and in synovial macrophages [155].

**Figure 17:**
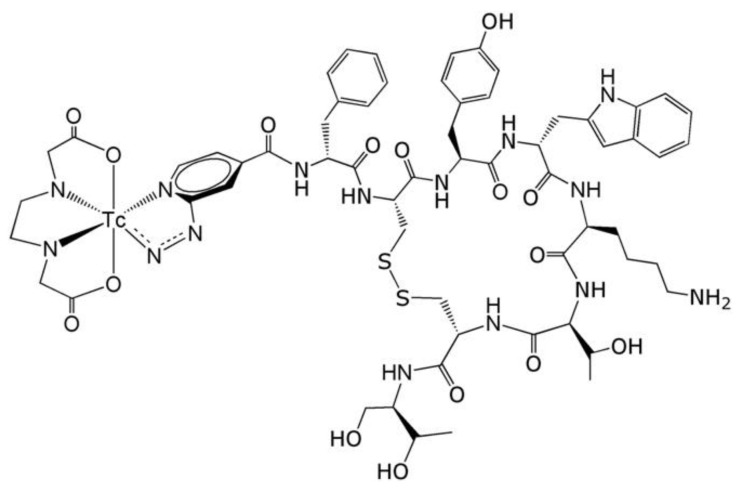
Structural image of [^99m^Tc]Tc-HYNIC-TOC. Reprinted from [166] with permission. Copyright 2021. Elsevier.

**Figure 18:**
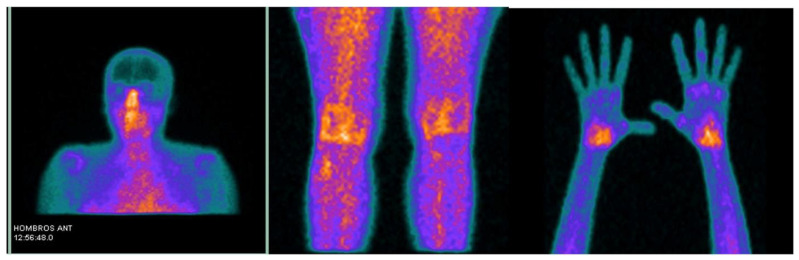
Uptake of [^99m^Tc]Tc-EDDA/HYNIC-TOC in the shoulders, submandibular glands, hands, and knees. Reprinted from open access source (CC BY-NC-ND 4.0) [165].

In one study, radiolabeled somatostatin [^99m^Tc]Tc-EDDA/tricine-HYNIC-tyr(3)-octreotide ([^99m^Tc]Tc-EDDA/HYNIC-TOC) (Figure 17) was used to study inflammation attenuation in the joint. Indeed, the high uptake of [^99m^Tc]Tc-EDDA/HYNIC-TOC was correlated with significant expression of somatostatin receptors, characterized by activation of endothelial cells and infiltration of lymphocytes in the synovium (Figure 18) [165].

### 2.6. Matrix Metalloproteinases

**Figure 19:**
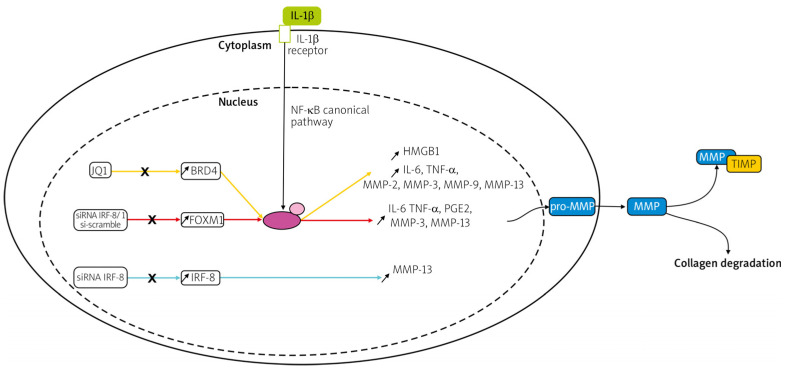
Pathways of Pro-MMP synthesis induced by IL-1β stimulation. Reprinted from open-access source (CC BY-NC-ND 4.0) [175].

Matrix metalloproteinases (MMPs) are zinc-containing, calcium-dependent endo-peptidases that are involved in degrading all kinds of ECM proteins, as well as in multiple cellular processes, such as cellular adhesion, differentiation, migration, proliferation, angiogenesis, host defense, and apoptosis [167,168,169,170,171,172]. MMPs are produced by macrophages, endothelial cells, leukocytes, synoviocytes, and chondrocytes and are essential for developmental and repair processes. Inflammatory cytokines stimulate the synthesis of MMPs (Figure 19) [173,174].

The most significant MMPs in RA are MMP-1, MMP-2, MMP-3, MMP-9, and MMP-13, which cleave fibrillar collagen and are correlated with VEGF and uPA. MMP-9 and MMP-13 work together to degrade joints [176,177,178,179,180].

**Figure 20:**
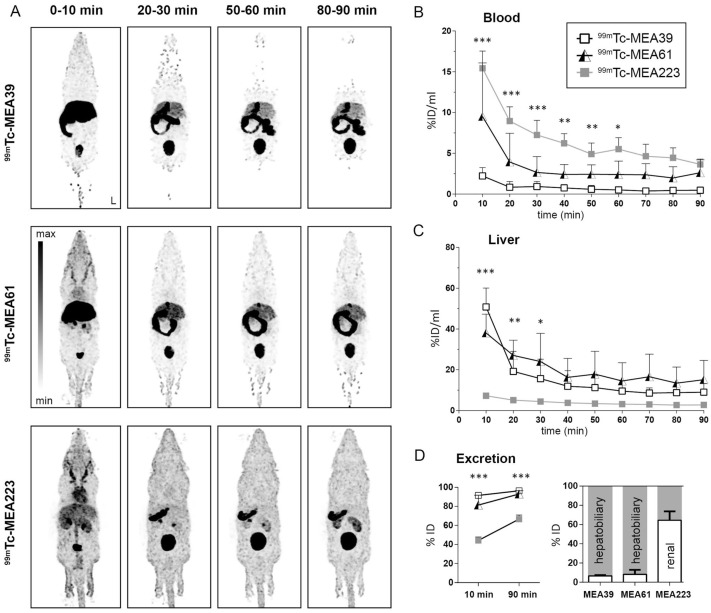
Investigation of the in vivo distribution and excretion of barbiturates following intravenous administration in adult C57BL/6 mice. (**A**) Biodistribution of the radiotracers [^99m^Tc]Tc-MEA39, [^99m^Tc]Tc-MEA61, and [^99m^Tc]Tc-MEA223. (**B**) The left ventricular volume of interest (VOI) was used to calculate dynamic in vivo blood radioactivity. (**C**) Hepatic accumulation of the radiotracer in vivo. (**D**) The relative proportion of the hepatobiliary and renal elimination pathways, as well as the radioactivity accumulation (shown as a percentage of the injected dosage), in the excretion organs (liver, gallbladder, intestine, kidney, and bladder) 10 and 90 min post-injection. The mean ± SD (n = 4–5) is displayed along with the ID (injected dose) and left image orientation (L). Statistical significance was determined via two-way ANOVA and Tukey’s post hoc test. Stars denote the significance of variations in [^99m^Tc]Tc-MEA223′s radiotracer uptake relative to that of the other compounds: * *p* < 0.05, ** *p* < 0.01, *** *p* < 0.001. Reprinted from open access source [181].

In one study, barbiturate-based MMP inhibitors labeled with technetium-99m (containing glycine ([^99m^Tc]Tc-MEA39), lysine ([^99m^Tc]Tc-MEA61), or the ligand HYNIC with the ionic ligand TPPTS ([^99m^Tc]Tc-MEA223]) were synthesized and evaluated in vitro and in vivo. Renal elimination was delayed only by [^99m^Tc]Tc-MEA223, which allows noninvasive imaging with high signal-to-noise ratios in a xenograft model (Figure 20) [181].

### 2.7. Tumor Necrosis Factor Alpha

**Figure 21:**
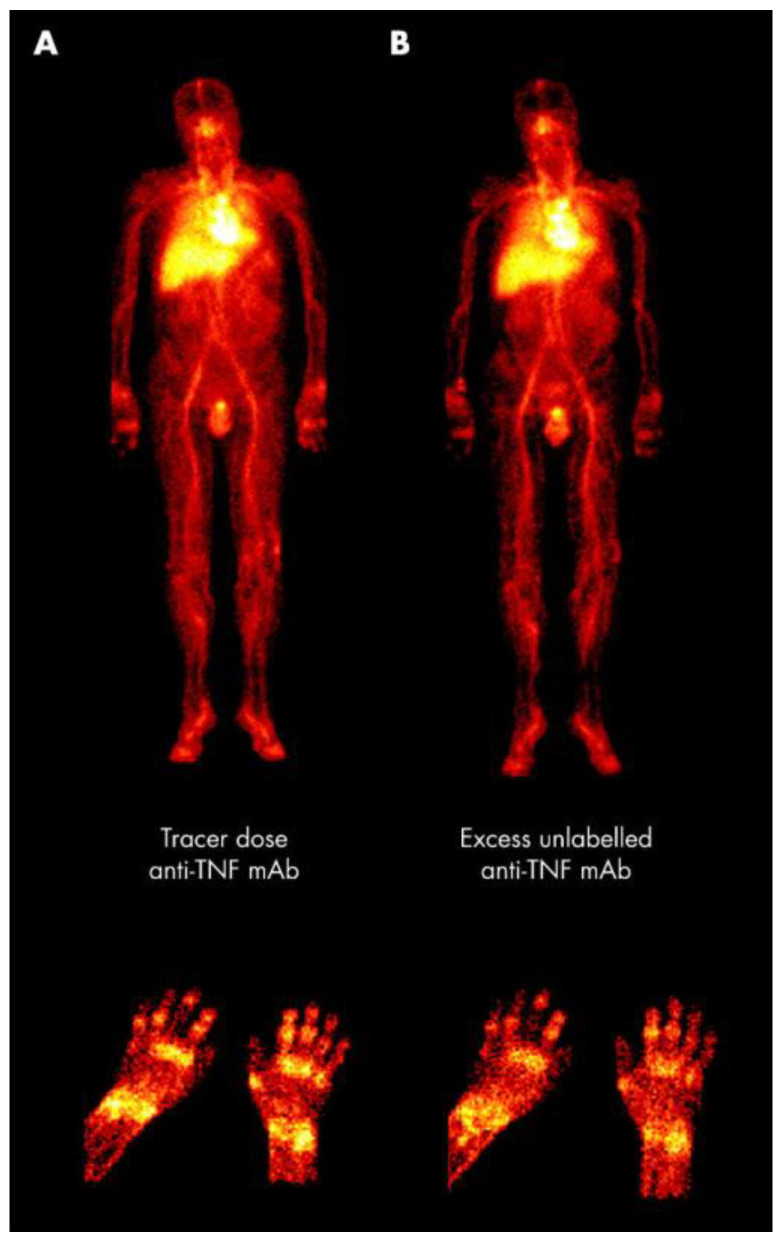
Scintigraphic images of an active RA patient’s hands and entire body after receiving an injection of [^99m^Tc]Tc-human-anti-TNF-mAb. (**A**) The initial imaging studies. (**B**) Following the injection of an excess of unlabeled anti-TNF mAb, there was a decrease in uptake in the joints (hands detail), but there was no change in uptake in the reticuloendothelial organs (whole body). Reprinted from open access source [189].

Tumor necrosis factor alpha (TNF-α) is a cytokine synthesized by monocytes, B cells, T cells, macrophages, and fibroblasts, which play a vital role in inflammation in RA patients [182]. TNF-α regulates various functions, such as activating ECs, synovial fibroblasts, and leukocytes, inducing chemokines, cytokines, adhesion molecules, and matrix enzymes, and also stimulates neovascularization via the angiopoietin 1 and angiopoietin 2 (Ang1/Ang2) Tie2-VEGF pathways [183,184,185,186,187,188]. Barrera et al. investigated the biodistribution and susceptibility of radiolabeled adalimumab ([^99m^Tc]Tc-adalimumab) in RA patients [189]. The aim of this study was to determine if this antibody could target TNF and visualize the synovitis. Each patient underwent two scintigraphic imaging scans: one before administration of [99mTc]Tc-adalimumab and the other after receiving non radiolabeled anti-TNF-mAb or an intramuscular corticosteroid injection. The [^99m^Tc]Tc-TNF-mAB uptake by joints decreased when non radiolabeled TNF-mAbs were injected simultaneously. Due to its retention, TNF-α was shown to be the radiolabeled mAb target in arthritic joints. The inflammation in the group that received corticosteroids had less radiopharmaceutical uptake. The authors concluded that [^99m^Tc]Tc-adalimumab is an effective radiopharmaceutical for detecting clinical changes in RA disease progression (Figure 21) [189].

A study by Malviya et al. was conducted to determine which radiopharmaceutical should be utilized for treatment. [^99m^Tc]Tc-infliximab and [^99m^Tc]Tc-adalimumab were injected to assess scintigraphic images of 12 and 9 patients with active RA, respectively. Imaging scans were performed before and three months after treatment with infliximab or systemic treatment with adalimumab. There were no changes observed in the biodistribution between the two radiopharmaceuticals. Scientigraphy of [^99m^Tc]Tc-human-immunoglobulin (HIG) were also performed on two patients. Although these two patients responded clinically, cold anti-TNF-mAb treatment did not affect [^99m^Tc]Tc-HIG uptake in the joints. It was concluded that [^99m^Tc]Tc-TNF-α was superior to [^99m^Tc]Tc-HIG [190,191].

**Figure 22:**
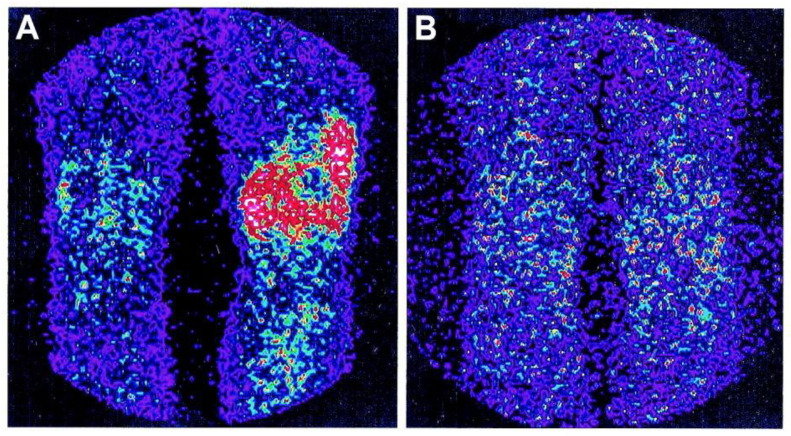
Scintigraphy images of [^99m^Tc]Tc-infliximab. (**A**) Before infliximab treatment. (**B**) 4 months after infliximab treatment. Reprinted from [192] with permission. Copyright 2005. John Wileys and Sons.

In another study, Conti et al. determined the level of TNF-mediated inflammation in the affected joints using a scintigraphic examination of a patient with RA. The patients received scintigraphic testing using ^99m^Tc-labeled infliximab 6 and 24 h after the injection of 555 MBq. As revealed by scintigraphy, a high level of intralesional TNF-α was indicated by the intensive build-up of [^99m^Tc]Tc-infliximab in the afflicted knee. Interestingly, there was no uptake in the inflammatory joint four months after intra-articular infliximab therapy (Figure 22) [192].

**Figure 23:**
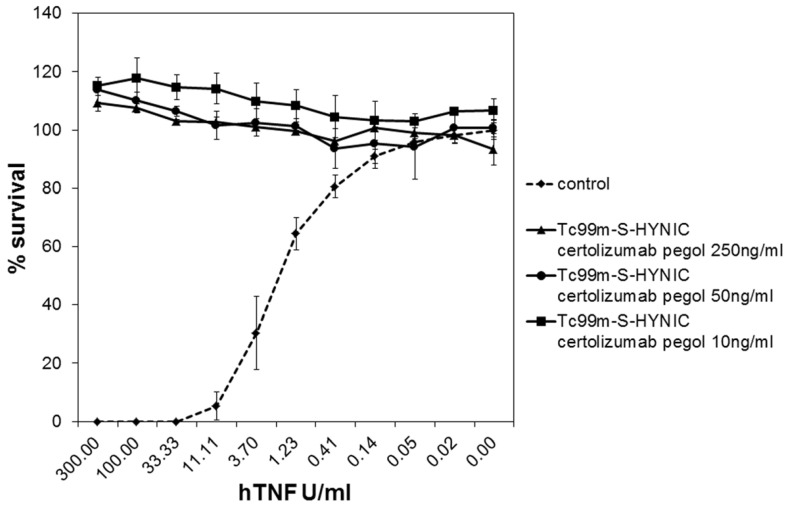
Treatment of TNF sensitive L929S cells with [^99m^Tc]Tc-S-HYNIC CZP. Reprinted from open access source [193].

In one study, the certolizumab pegol (CZP), was labeled with [^99m^Tc]Tc-S-HYNIC to assess its ability to neutralize TNF-induced cytotoxicity using L929S cells. The formulation effectively blocked TNF activity at low concentrations (10 ng/mL), showing comparable effects to unlabeled CZP, with or without the HYNIC linker. Infliximab was used as a positive control, also displayed effective neutralizing activity, albeit less potent at the given doses. These findings suggest a promising radiolabeling approach for CZP, indicating potential applications in TNF-targeted imaging and therapy for inflammatory diseases such as spondyloarthritis (SpA) and RA (Figure 23) [193].

**Figure 24:**
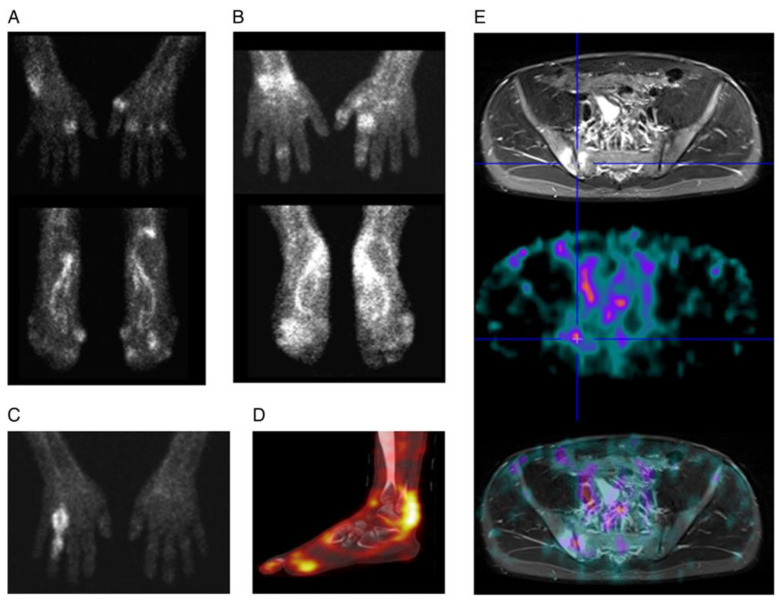
Certolizumab pegol labeled with Tc99m was distributed in the hands, feet, and sacroiliac joints 4-5 h after injection. (**A**) A patient with active rheumatoid arthritis exhibited a polyarticular pattern of joints without involvement of distal interphalangeal joint (DIP). (**B**) Left second digit distal interphalangeal joint uptake in polyarticular psoriatic arthritis patients. (**C**) Tracer uptake in dactylitis patient of the fourth digit. (**D**) SPECT-CT scan of a patient’s right foot showing Achilles tendon enthesitis and spondyloarthritis. (**E**) Fusion of sacroiliac joint SPECT and MRI images in an axial spondyloarthritis patient. Reprinted from open access source (CC BY-NC-ND 4.0) [194].

In another study, a radiolabeled ^99m^Tc, certolizumab CZP, was coupled with succinimidyl-6-hydrazino-nicotinamide. Three-time points were used to obtain whole-body images and images of the hands, feet, and sacroiliac joints in patients (SpA and RA). Semiquantitative scoring was applied to the immunoscintigraphic results and CZP was subsequently used to treat each patient. The scintigraphy-positive group had significantly more clinically afflicted joints or abnormal US findings in peripheral joints (*p* < 0.001). Bone marrow edema was found more frequently (*p* < 0.001) in magnetic resonance imaging (MRI) quadrants with tracer uptake in patients of SpA. At the patient level, joints with evident tracer uptake decreased probability of remaining in pain after 24 weeks of treatment with CZP (OR = 0.42, *p* = 0.04) compared with joints with no tracer uptake (Figure 24) [194].

## 3. Conclusions and Future Perspectives

RA is a systemic, autoimmune, inflammatory disorder that damage cartilage and joints. There has been significant advancement in the use of ^99m^Tc as a targeted radiopharmaceutical for molecular imaging of RA. ^99m^Tc SPECT/CT provides a sophisticated diagnostic method with increased accuracy due to accurate anatomical localization.

The focus on ^99m^Tc SPECT in hybrid imaging has emerged as a notable breakthrough in RA diagnosis. This review focused on the use of ^99m^Tc labeled targeting vectors for RA diagnosis, emphasizing the importance of SPECT in providing functional and molecular imaging of affected joints with RA. The short half-life and low toxicity of ^99m^Tc make it an ideal choice for targeted imaging of joints affected by RA.

The application of ^99m^Tc in RA diagnosis holds great potential for further research and clinical implementation. Future studies could confirm or explore novel ligands and targeting vectors to increase the specificity and sensitivity of ^99m^Tc-labeled agents, thereby improving the visualization of molecular processes related to inflammation, angiogenesis, and bone turnover in RA-affected joints. Furthermore, improvements in instrumentations, imaging technology, and the use of artificial intelligence may enhance the qualities of ^99m^Tc SPECT, enabling even more precise and thorough evaluations.

Collaborations between clinicians, researchers, and specialists in molecular imaging may result in the development of radiolabeled targeting vectors for personalized medicine for the accurate and customized treatment of RA, as well as for monitoring treatment responses and early disease progression assessment.

In summary, using ^99m^Tc labeled targeting vectors unveils new paths for innovative strategies in diagnosis as well as understanding the complexities of RA. ^99m^Tc applications associated with advancements in molecular imaging, hold great promise for improving RA diagnosis and management, ultimately enhancing patient outcomes.
